# Enuresis, ADHD and BDNF: A Narrative Review of the Hypothesized Interconnections and Potential Triplet Relationship

**DOI:** 10.3390/brainsci16040372

**Published:** 2026-03-29

**Authors:** Maria Milioudi, Stella Stabouli, Dimitrios Zafeiriou, Efthymia Vargiami

**Affiliations:** 1st Pediatric Department, Faculty of Health Sciences, School of Medicine, Aristotle University of Thessaloniki, 54642 Thessaloniki, Greece; sstaboul@auth.gr (S.S.); dizafeir@auth.gr (D.Z.); evargiam@auth.gr (E.V.)

**Keywords:** attention-deficit/hyperactivity disorder, enuresis, brain–derived neurotrophic factor, neurotrophic factors, primary monosymptomatic nocturnal enuresis, lower urinary tract symptoms, daytime urinary incontinence, central nervous system, proBDNF, locus coeruleus

## Abstract

**Highlights:**

**What are the main findings?**

- Current evidence suggests possible interconnections between ADHD, enuresis and BDNF.
- BDNF contributes to bladder pathophysiology and neurodevelopmental dysfunction, providing a plausible biological basis for further investigation.
- Narrative synthesis was conducted to summarize the current state of knowledge regarding the three terms under consideration.

**What are the implications of the main findings?**

- BDNF represents a candidate molecule worthy of further study in the context of ADHD and enuresis.
- This review identifies significant gaps in the literature and proposes directions for future research to clarify potential mechanistic links.

**Abstract:**

Attention-deficit/hyperactivity disorder (ADHD), brain–derived neurotrophic factor (BDNF), and enuresis are interconnected in several ways, primarily through their potential links to neurodevelopmental factors and brain function. ADHD is considered a neurobehavioral and neuropsychiatric condition characterized by numerous comorbidities, and it represents one of the most frequently encountered neuropsychiatric disorders in clinical practice. Enuresis constitutes a subgroup of intermittent incontinence occurring during sleep that can be further subdivided into monosymptomatic (MNE) and non-monosymptomatic enuresis (NMNE). BDNF plays a crucial role in neurodevelopment, including neuronal growth, proliferation, survival, differentiation, and synaptic plasticity. This narrative review synthesized available literature identified through a systematic search of PubMed/MEDLINE, Science Direct, and Scopus databases (January 2000–December 2025). However, the evidence base is heterogeneous, and findings regarding BDNF in ADHD are inconsistent. Studies examining BDNF in enuresis often involve urinary BDNF, which reflects local bladder production rather than central BDNF activity. Further research is needed to clarify the specific roles of BDNF in the development and manifestation of these conditions and to fully elucidate the complex relationship between BDNF, ADHD, and enuresis.

## 1. Introduction

Enuresis and attention deficit hyperactivity disorder (ADHD) frequently co-occur, with research indicating a higher prevalence of enuresis in individuals with ADHD [1,2]. Brain-derived neurotrophic factor (BDNF) is a protein involved in brain development and function [3,4]. Several studies have explored potential links between BDNF and both ADHD and enuresis [5,6,7].

ADHD, BDNF and enuresis are interconnected in several ways, primarily through their potential links to neurodevelopmental factors and brain function. The hypothesized mechanistic pathways linking these three domains include: (1) shared neurodevelopmental origins affecting brainstem arousal systems [8]; (2) involvement of the locus coeruleus and pontine micturition center, both of which are modulated by BDNF [8,9]; (3) the role of BDNF in noradrenergic and dopaminergic system development [9,10]; and (4) BDNF effects on bladder sensory neurons and central micturition control [11,12]. However, direct evidence for a unified three-way mechanism remains limited.

**Figure 1:**
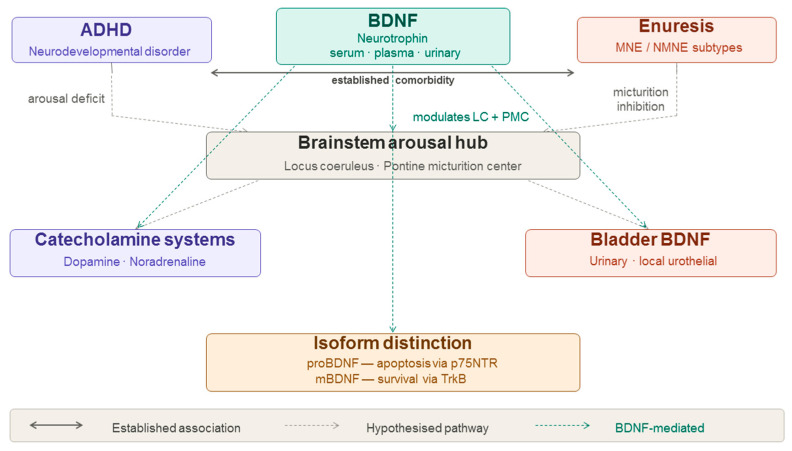
Schematic representation of the hypothesized mechanistic relationships among ADHD, enuresis, and BDNF. Solid bidirectional arrows indicate the well-established clinical comorbidity between ADHD and enuresis. Dashed arrows denote mechanistically plausible but not yet directly demonstrated pathways. Green dashed arrows indicate BDNF-mediated connections. LC, locus coeruleus; PMC, pontine micturition center; MNE, monosymptomatic nocturnal enuresis; NMNE, nonmonosymptomatic nocturnal enuresis; proBDNF, precursor BDNF; mBDNF, mature BDNF; p75NTR, p75 neurotrophin receptor; TrkB, tropomyosin receptor kinase B.

This review aimed to address a gap in the literature by integrating three complementary lines of evidence into a coherent whole. These three terms have largely been investigated separately, with limited attempts to examine their interrelationships in the pediatric population, despite several studies indicating their likely interrelated and mutually influential nature. This review aimed to elucidate the possible interconnections among the three factors and to identify directions for future research. To this end, we provide a brief overview of each topic, followed by pairwise analyses of the literature linking ADHD with enuresis, ADHD with BDNF, and enuresis with BDNF. The hypothesized mechanistic pathways linking these three domains are summarized in Figure 1.

## 2. Search Strategy and Study Selection

This study is a narrative review with a systematically conducted literature search aimed at exploring existing evidence on potential interconnections among ADHD, enuresis, and BDNF in the pediatric population. A comprehensive search was conducted across PubMed/MEDLINE, Science Direct and Scopus databases. The search was restricted to human, English-language studies published between January 2000 and December 2025. Keywords used included “ADHD”, “Enuresis”, “BDNF”, “Child”, “Children”, “Pediatric” and “NGF”, subsequently combined in pairs to maximize sensitivity. An additional manual search of the reference lists of eligible articles was performed to identify studies not captured by the database search.

Studies were considered eligible if they reported original research in participants aged 0–18 years, with ADHD diagnosed according to standardized criteria (DSM-IV, DSM-5, or ICD-10/11). Studies were excluded if they focused exclusively on adult populations, did not distinguish pediatric from adult data, involved animal subjects, or were not available as full-text publications in English. Case reports, editorials, letters, conference abstracts, and studies examining therapeutic interventions were also excluded, as these fell outside the scope of the current work. Following screening of 173 identified records, 30 studies were included in the final narrative synthesis: 9 addressing ADHD and enuresis, 6 addressing ADHD and BDNF, and 15 addressing BDNF and enuresis.

Importantly, although the above eligibility criteria were applied to studies examining the ADHD-enuresis and ADHD-BDNF relationships, the literature on BDNF and enuresis is limited and studies in this area often do not require an ADHD diagnosis as an inclusion criterion. Relevant studies on BDNF in enuresis, with or without ADHD comorbidity, were therefore cautiously included, with this heterogeneity explicitly noted and extrapolation to the full triad limited accordingly. A conceptual figure illustrating the hypothesized mechanistic pathways is provided as Figure 1, and a summary of all 30 included studies is provided in Appendix A Table A1. Generative AI was not used for data collection, analysis, or any other purpose in this review.

## 3. Literature Review

### 3.1. Attention Deficit Hyperactivity Disorder

ADHD is a neurodevelopmental disorder characterized by developmentally inappropriate levels of hyperactivity, impulsivity, or inattention that interfere with daily functioning or development (American Psychiatric Association, 2013). The Diagnostic and Statistical Manual of Mental Disorders, fifth edition (DSM-5), published in 2013, provides the criteria for diagnosing ADHD and specifies two subgroups: predominantly inattentive (ADHD-I) and predominantly hyperactive-impulsive (ADHD-H). A combined subgroup (ADHD-C) exhibiting characteristics of both presentations has also been described. The DSM-5 reclassified ADHD under neurodevelopmental disorders, recognizing its onset and developmental course. An ADHD diagnosis requires at least six symptoms in children up to age 16, whereas adolescents (17+) and adults must present with at least five symptoms from either the inattention or hyperactivity–impulsivity categories. Symptoms, which are expected to interfere with social, academic, or occupational functioning, should be present before the age of 12 and occur in multiple settings, such as home, school, extracurricular activities, or sports. Salari et al., in a recent global meta-analysis, found that the general prevalence of ADHD in children aged under 12 years was 7.6%, whereas in adolescents (12–18 years) it was 5.6% [13].

ADHD is considered a neurobehavioral and neuropsychiatric condition characterized by numerous comorbidities and is among the most prevalent neurodevelopmental disorders encountered in the pediatric population. Notably, children with epilepsy show increased vulnerability to ADHD, with prevalence rates of 29.1–38% [14], suggesting shared neurobiologic mechanisms [15]. ADHD frequently co-occurs with other disorders in children, most commonly oppositional defiant disorder, other behavioral or conduct problems, anxiety disorders, and learning disabilities [16]. Depression, autism spectrum disorder, tic disorders, and enuresis can also be comorbid. These additional conditions can exacerbate ADHD symptoms and functional impairment, highlighting the importance of comprehensive assessment and treatment [17]. Multiple genetic and environmental factors contribute to ADHD risk, including prenatal exposures and perinatal complications, though causality requires further investigation [18].

ADHD is linked to academic underachievement, difficulties with cognitive tasks, executive function impairment, poor planning, working memory deficits, impaired inhibitory control, and reduced alertness, all of which undermine the learning process [19]. Depending on symptom severity, interventions include stimulant medications as well as occupational and psycho-educational remediation programs despite the debatable evidence base for the latter [20].

### 3.2. Enuresis

Enuresis is a common, multifactorial condition with a genetic basis, exhibiting considerable heterogeneity and complexity depending on the specific features of each patient. According to the International Children’s Continence Society (ICCS), enuresis is involuntary micturition during sleep that occurs after the age at which bladder control is typically expected, usually in children aged over five years. Consistent with ICD-10 and DSM-V criteria, the symptom of incontinence requires a minimum age of 5 years, at least one episode per month, and a minimum duration of 3 months to qualify as a clinical condition. Additionally, enuresis can be qualified as frequent (>4 episodes per week) or infrequent (<4 episodes per week). The term enuresis should not be used to refer to daytime incontinence. Thus, enuresis is considered a subgroup of intermittent incontinence during sleep and can be further subdivided into monosymptomatic (MNE) and nonmonosymptomatic enuresis (NMNE).

Children with MNE experience urinary incontinence exclusively at night, with no other urogenital or gastrointestinal tract symptoms. NMNE, also referred to as polysymptomatic nocturnal enuresis (PolyNE), is a type of bedwetting in children accompanied by daytime lower urinary tract symptoms (LUTS) such as urgency, daytime incontinence, hesitancy or straining, a weak urine stream or dribbling, painful urination or dysuria, and holding maneuvers. Both MNE and NMNE can be further separated into two subgroups: primary and secondary enuresis. Primary enuresis refers to a child who has never achieved a consistent period of at least 6 months of nighttime dryness. When organic causes are ruled out, the condition is considered primary (or nonorganic) enuresis, often associated with genetics, maturational delay in bladder control, sleep arousal issues, or nocturnal polyuria. Secondary enuresis refers to a child who experiences bedwetting after being dry during the day and night for at least 6 to 12 months, often triggered by psychological stress from life changes or an underlying medical condition such as a urinary tract infection, diabetes, constipation, or a sleep disorder [21].

Previous studies estimate the prevalence of enuresis at 9–12%, with an annual spontaneous resolution rate of up to 15%. Thus, enuresis may resolve spontaneously without treatment [22]. Among children with enuresis, 80–90% present with primary enuresis, in which bedwetting is largely influenced by genetics, miscommunication between the brain and bladder, nocturnal polyuria (excessive nighttime urine production), and low bladder capacity. Primary enuresis is also associated with sleep arousal disorders (difficulty waking to a full bladder), maturational delays in bladder control or hormone production (such as antidiuretic hormone or ADH), and sometimes constipation [23].

The pathophysiology and etiology of enuresis represent a multifactorial process, incorporating psychological, biological, developmental, and environmental factors, as well as suspected genetic influences. Although genetics plays a strong role, no clear genotype–phenotype correlation has been established. Existing literature links enuresis to loci on chromosomes 8, 12q, 13q, and 22, demonstrating notable locus heterogeneity [24]. As research on risk genes advances, new loci of interest continue to emerge. For instance, PRDM13 and EDNRB participate in established pathophysiological mechanisms of nocturnal enuresis, while SIM1 influences the production of arginine vasopressin in the hypothalamus. These genes may contribute heterogeneously to processes related to sleep, urine production, and excretion, ultimately guiding potential therapeutic interventions [25].

From a neurobiological perspective, MNE is particularly relevant to BDNF research because its pathophysiology primarily involves central mechanisms, including brainstem arousal systems [8], pontine micturition centers [8], and sleep regulation [23], in which BDNF contributes to neuronal survival, synaptic plasticity, and neurotransmitter modulation [3,26,27]. In contrast, NMNE primarily involves peripheral bladder dysfunction, in which urinary BDNF may reflect local neuroplastic changes rather than central BDNF activity [7,11,12,28,29].

### 3.3. Brain–Derived Neurotrophic Factor

BDNF plays a crucial role in neurodevelopment, including neuronal growth, survival, differentiation, and synaptic plasticity. BDNF was originally purified from pig brain tissue in 1982 and was shown to have immunogenic properties similar to, yet distinct from, nerve growth factor (NGF), which had been previously characterized [30] (an animal/in vitro study retained for its seminal characterization of BDNF; see Appendix A Table A1). Italian neurodevelopmental biologist Rita Levi-Montalcini and American biochemist Stanley Cohen received the 1986 Nobel Prize in Physiology or Medicine for their pivotal discoveries of NGF and epidermal growth factor (EGF), respectively. Their research in the 1950s laid the groundwork for identifying the broader family of neurotrophins.

Neurotrophic factors can be grouped into three main families: neurotrophins (BDNF, NGF, neurotrophin-3, neurotrophin-4), the CNTF family (CNTF, leukemia inhibitory factor, interleukin-6, prolactin, growth hormone, leptin, interferon-α,β,γ, oncostatin M), and the GDNF family (GDNF, artemin, neurturin, and persephin). Further analysis of these factors lies beyond the scope of the present review. BDNF synthesis involves multiple sequential isoforms. The 247-amino acid BDNF protein is initially produced in the endoplasmic reticulum as preproBDNF, and then transported to the Golgi apparatus, where proBDNF is generated. Proteolytic cleavage of proBDNF forms produces the mature isoform, mBDNF [26].

BDNF signals primarily through tropomyosin receptor kinase B (TrkB), a high-affinity tyrosine kinase essential for the growth, development, and synaptic plasticity of glutamatergic and GABAergic neurons. This signaling supports critical functions such as learning and memory. BDNF also interacts with the low-affinity p75 neurotrophin receptor (p75NTR), a nonspecific receptor for all neurotrophins, which plays a lesser role in neuronal signaling. By affecting neuronal differentiation, BDNF modulates neurotransmission within serotonergic and dopaminergic circuits. BDNF exhibits paracrine and autocrine effects in pre- and postsynaptic regions, facilitating synaptic consolidation by transforming transient synaptic activity into long-term memory. It plays a central role in both the functional and structural formation of hippocampal dendritic spines, a process that continues into adulthood, contributing to neurogenesis and synaptic plasticity. Alterations in adult neurogenesis or spine density can lead to learning and memory deficits and may contribute to depression-like symptoms [3].

The *BDNF* gene is located on the short arm of human chromosome 11, specifically within the 11p13-14 band. Evidence indicates that *BDNF* function is associated with the estimated volume of the prefrontal cortex in both humans and animal models [31]. Pan et al. demonstrated that BDNF crosses the blood-brain barrier bidirectionally, with an efflux rate similar to the cerebrospinal fluid reabsorption rate, exhibiting generally congruent fluctuations in brain and serum levels [32]. BDNF is highly concentrated in the brain, particularly in the hippocampus, amygdala, and cerebral cortex. Notably, serum BDNF primarily reflects platelet stores and is not interchangeable with plasma BDNF. Serum concentrations are approximately 20 times higher than plasma, and the two compartments are not correlated [33]. Plasma BDNF levels correlate to some degree with platelet BDNF levels [34] and may better represent freely circulating BDNF, whereas urinary BDNF likely reflects local production by the bladder urothelium [7,11,12,28,29]. Although blood BDNF levels can serve as a useful proxy for brain health and resilience, considerable variability in measurements and clinical interpretation exists across studies. Evidence from adult populations, such as decreased serum BDNF levels in adults with depression [35], cannot be generalized to children with ADHD or enuresis without direct pediatric validation.

Converging evidence suggests that mBDNF bioactivity can counteract the actions of proBDNF. The equilibrium between proBDNF and mBDNF regulates several CNS functions, although the precise causal pathways remain unclear [27]. Furthermore, proBDNF and mBDNF often exert opposing biological effects: proBDNF promotes apoptosis via p75NTR, whereas mBDNF supports neuronal survival through TrkB receptors. Most clinical studies, however, measure total BDNF without distinguishing isoforms, representing a significant limitation [27].

## 4. Possible Correlations in Pairs

The proposed interconnections among ADHD, enuresis, and BDNF are illustrated in Figure 1.

### 4.1. Attention Deficit Hyperactivity Disorder and Enuresis

The association between ADHD and enuresis is well-documented, although the underlying mechanisms remain under investigation. According to data from the National Institutes of Health (NIH), 28–40% of individuals with ADHD also experience enuresis. Two main CNS functional impairments have been proposed in the etiology of nocturnal enuresis. First, bladder-originating stimuli are not efficiently transmitted and processed during sleep, failing to elicit the expected arousal response. Second, inhibition of the urinary reflex is insufficient during sleep, resulting in bedwetting. Both mechanisms are regulated by brainstem nuclei: the locus coeruleus, which modulates attention, arousal, sleep-wake cycles, and the fight-or-flight response, and the lateral region of the pontine micturition center (PMC), dysfunction of which leads to impaired inhibition of micturition [8] (a pre-2000 commentary retained for its mechanistic relevance; see Appendix A Table A1).

ADHD arises from atypical development of brain regions such as the prefrontal cortex, basal ganglia, and anterior cingulate cortex, which govern executive functions essential for self-regulation. Locus coeruleus (LC) noradrenergic neurons provide the primary source of noradrenaline in the brain, originating in the brainstem and projecting throughout the central nervous system. These neurons exhibit diverse electrophysiological properties, form distinct functional ensembles, and contribute to processes such as pain and anxiety. They also support hypotheses linking ADHD, sleep, and BDNF, although a detailed discussion of these mechanisms is beyond the scope of this review. Some studies have suggested that enuresis may negatively affect cognitive functions, including IQ and visuospatial skills, in children with ADHD [36].

The strength of the association between ADHD and enuresis varies across studies. Population-based studies have provided more reliable estimates; for example, Shreeram et al. [1] examined 8256 children in the United States and found significant associations between enuresis and ADHD. However, confounding factors such as socioeconomic status, family history, and comorbid psychiatric conditions may influence this relationship [1,37]. Ascertainment bias in clinical samples may further overestimate comorbidity rates compared with population-based studies [2]. Although arousal deficits and brainstem mechanisms offer a plausible shared pathway involving the locus coeruleus and pontine micturition center [8] (a pre-2000 commentary included for its mechanistic relevance; see Appendix A Table A1), alternative explanations include medication effects, psychological distress secondary to enuresis exacerbating ADHD symptoms, and shared genetic factors [24,25].

Although most studies focus on the association between nocturnal enuresis and ADHD, attention should also be given to daytime urinary incontinence (DUI), as its cooccurrence with ADHD is more common. When any type of incontinence coexists with ADHD, evidence-based guidelines recommend concurrent treatment of both conditions [2]. In routine clinical practice, children with all types of incontinence should be screened for concurrent psychological symptoms, such as ADHD or autism spectrum disorder, and vice versa, using standardized, validated questionnaires [1,37].

### 4.2. Attention Deficit Hyperactivity Disorder and Brain-Derived Neurotrophic Factor

Evidence suggests BDNF involvement in ADHD neurobiology, with some studies reporting differences in BDNF levels between affected individuals and controls, although findings are inconsistent. The pathophysiology of ADHD is partly attributed to dysregulation of catecholaminergic systems and frontostriatal and frontocerebellar circuits, prompting investigation of genes that regulate dopamine and epinephrine signaling. Other potentially affected regions include the prefrontal cortex, temporal and parietal cortical areas, basal ganglia, and the cerebellum [38]. BDNF is essential for the survival and maturation of midbrain dopaminergic neurons [10] and for the phenotypic diversification of locus coeruleus noradrenergic neurons [9]. Cho et al. demonstrated that estrogen can modulate BDNF expression in a sex-specific manner in children with ADHD. In addition, reproductive hormones act on cortical neurons that produce BDNF, altering its functionality [39].

Specifically, some studies have reported increased BDNF levels in children with ADHD compared with controls [5,40], whereas others found decreased levels [6] or no significant difference [41,42]. This heterogeneity likely reflects multiple factors, including differences in age groups [5], medication status [42], ADHD subtypes [40], and the compartment measured (serum vs. plasma) [39,41]. Tsai reported that decreased BDNF levels in children with ADHD may occur either as a primary feature contributing to the developmental deficits of ADHD or secondarily due to neuronal dysfunction arising from these developmental deficits.

The hypothesis that BDNF dysregulation underlies ADHD is reinforced by accumulating evidence of its role in the development and regulation of the dopamine system [6] (a theoretical hypothesis paper without original data; see Appendix A Table A1). Shim et al. reported higher BDNF levels in patients with ADHD compared with controls, suggesting compensatory upregulation in response to dysregulated dopaminergic and serotonergic pathways. However, this effect appears less pronounced in younger children. Elevated BDNF levels show a positive correlation with the severity of inattention symptoms [5,40]. Furthermore, reduced serum BDNF levels have been observed in adult patients with ADHD compared with healthy controls, suggesting that BDNF dysregulation may persist into adulthood [43].

BDNF levels in humans increase following exercise, with the magnitude of change influenced by training intensity. This effect may extend to children with ADHD, who are characteristically hyperactive [44]. Scassellati et al. reported no significant differences in serum BDNF levels in drug-naïve children with ADHD. Such discrepancies likely reflect the intrinsic heterogeneity of ADHD, the complexity of its pathophysiology, differences in blood sampling methods, laboratory techniques, and genetic background [41,42].

Critically, current evidence does not support BDNF as a diagnostic biomarker for ADHD. The marked heterogeneity across studies precludes definitive conclusions. At best, BDNF may represent one of many molecules involved in ADHD pathophysiology, but its clinical utility remains unproven [41].

### 4.3. Enuresis and Brain-Derived Neurotrophic Factor

Several studies have explored potential links between BDNF and enuresis, examining whether BDNF concentrations or genetic variants are associated with the condition, with particular focus on urinary BDNF levels. BDNF is the most abundant neurotrophin in the brain and promotes the growth, maturation, and survival of both the central and peripheral nervous systems. It also modulates neurotransmission and contributes to mechanisms of neuronal plasticity, including long-term potentiation and learning [4].

BDNF is expressed in the bladder and kidney, in addition to the brain. Bladder concentrations are approximately 15 times higher than those in the cerebral parenchyma, suggesting an important local role in bladder sensory and motor neuron function [11]. Elevated production of neurotrophic factors in bladder dysfunction, arising from spinal cord injury, inflammation, or detrusor overactivity, reflects neuroplastic changes in sensory afferents rather than central BDNF activity [12]. Urinary BDNF has been proposed as an objective biomarker for overactive bladder, with levels appearing more sensitive than urinary NGF in affected females [28].

Ece et al. reported that elevated urinary neurotrophin levels in children with PMNE may reflect delayed neuromaturation or increased local bladder production, indicating heightened bladder sensory nerve excitability and supporting enuresis development [7]. Morizawa et al. reported that urinary NGF/Cr and BDNF/Cr ratios were significantly higher in children with MNE compared with healthy controls, proposing urinary NGF/Cr as a predictive marker for unsatisfactory treatment outcomes [29].

The hypothesis that BDNF contributes to enuresis through effects on bladder sensory neurons [12] or pontine micturition centers [8] is mechanistically plausible. However, as no studies have directly examined these pathways in humans, the hypothesis remains speculative. As urinary BDNF reflects local bladder urothelial production rather than central BDNF activity, these findings do not establish a systemic or neurodevelopmental BDNF deficit and should not be extrapolated to central mechanisms without direct evidence.

## 5. Conclusions

This narrative review summarizes preliminary evidence suggesting potential interconnections among ADHD, enuresis, and BDNF. Although the comorbidity between ADHD and enuresis is well-established clinically, and both conditions involve brain regions and neurotransmitter systems in which BDNF plays key physiological roles, direct evidence supporting BDNF as a shared biological mediator remains limited, indirect, and methodologically heterogeneous.

Current evidence does not support BDNF as a clinical biomarker for diagnosis, prognosis, or treatment monitoring in either condition. Substantial variability across studies, likely attributed to differences in age groups, measurement methods, clinical phenotypes, and BDNF compartments (serum, plasma, and urine), precludes definitive conclusions. Importantly, urinary BDNF, frequently examined in enuresis research, reflects local bladder urothelial production rather than central BDNF activity and should not be conflated with systemic or central measurements.

Furthermore, most clinical studies assess total BDNF without distinguishing between isoforms. Given that proBDNF promotes apoptosis, whereas mBDNF supports neuronal survival, treating BDNF as a single entity represents a significant methodological limitation that future research should address.

Based on these findings, we propose the following testable hypotheses for future research:

**Hypothesis** **1.** *Central BDNF deficiency: Children with both ADHD and enuresis may show lower serum/plasma BDNF levels than those with either condition alone, if central BDNF deficiency drives the comorbidity.*

**Hypothesis** **2.** *Enuresis subtype specificity: Urinary BDNF may be elevated specifically in NMNE (with daytime LUTS) but not in isolated MNE, reflecting peripheral bladder neuroplasticity rather than central dysfunction.*

**Hypothesis** **3.** *Genetic moderation: The BDNF Val66Met polymorphism may moderate the strength of association between ADHD and enuresis, and should be examined across enuresis subtypes.*

**Hypothesis** **4.** *Longitudinal trajectory: Longitudinal studies should examine whether BDNF levels change with age, enuresis resolution, and treatment response, accounting for ADHD subtype and medication status.*

Testing these hypotheses rigorously will require methodological standardization. Future studies should: (1) measure and report BDNF compartments (serum, plasma, urinary) and isoforms (proBDNF vs. mBDNF) separately; (2) prespecify enuresis subtype (MNE vs. NMNE) and ADHD presentation as stratification variables; and (3) control for key confounders, including age, sex, stimulant medication, and comorbid conditions. Collaborative, multicenter designs with standardized protocols will be essential to achieve adequate sample sizes for meaningful subgroup analyses.

Until such evidence emerges, the hypothesis that BDNF represents a shared biological link between ADHD and enuresis remains compelling but unproven. This review identifies these gaps as a priority for future research and provides a framework for subsequent mechanistic and clinical investigations.

## Data Availability

No new data were created or analyzed in this study. Data sharing is not applicable to this article.

## Abbreviations

The following abbreviations are used in this manuscript:

ADHD	Attention-deficit hyperactivity disorder
ADHD-I	ADHD inattentive type
ADHD-H	ADHD hyperactive and impulsive type
ADHD-C	ADHD combined type
DSM-5	Diagnostic and Statistical Manual of Mental Disorders (fifth edition)
NTF	Neurotrophic factor
CTNF	Ciliary neurotrophic factor
BDNF	Brain-derived neurotrophic factor
GDNF	Glial cell line-derived factor
NGF	Nerve growth factor
Trk	Tyrosine kinase receptor
MNE	Monosymptomatic enuresis
NMNE	Non-monosymptomatic Enuresis
PMNE	Primary monosymptomatic nocturnal enuresis
LUTS	Lower urinary tract symptoms
DUI	Daytime urinary incontinence
PMC	Pontine micturition center
LC	Locus coeruleus

## Appendix A

**Table A1:** Summary of all 30 studies included in the narrative review, organized by thematic section.

Author, Year [Ref]	Study Design & N	Population & Enuresis Subtype	BDNF Compartment	Main Findings	Key Limitations
**Section A—ADHD and Enuresis (n = 9)**					
Dossche et al., 2016 [23]	Review	Children with MNE; MNE subtype	N/A	Nocturnal polyuria, low bladder capacity, and sleep arousal disorder are central mechanisms of MNE; circadian renal physiology implicated	Review format; focuses exclusively on MNE; no original data
Jørgensen et al., 2021 [25]	Genome-wide association study; large population cohort	Enuresis populations; subtype not specified	N/A	*PRDM13*, *EDNRB*, and *SIM1* gene loci identified; SIM1 implicated in vasopressin pathway relevant to nocturnal polyuria	GWAS limitations; replication needed; no ADHD arm
Koff, 1996 [8] †	Review/commentary	Children with nocturnal enuresis; subtype not specified	N/A	Locus coeruleus and pontine micturition center proposed as key brainstem nuclei in arousal and micturition inhibition deficits in enuresis	Pre-2000 commentary; mechanisms not directly tested in humans; no BDNF data; borderline exclusion criterion
von Gontard & Equit, 2015 [2]	Review	Children with ADHD and incontinence; MNE and DUI subtypes	N/A	Synchronous therapy of ADHD and incontinence recommended; DUI cooccurs more frequently with ADHD than nocturnal enuresis; evidence-based guidelines reviewed	Review format; clinical focus; no biomarker data
von Gontard et al., 2011 [37]	Review	Children with urinary and fecal incontinence; mixed subtypes	N/A	Routine screening for ADHD in children with incontinence recommended; bidirectional relationship proposed; validated questionnaires discussed	Review format; psychiatric focus; no original data; no BDNF
Shreeram et al., 2009 [1]	Population-based cross-sectional study; N = 8256	Nationally representative U.S. children; enuresis subtype not specified	N/A	Significant association between enuresis and ADHD confirmed in nationally representative sample; strongest epidemiological evidence for comorbidity in review	Cross-sectional; parent-reported data; enuresis subtype not distinguished; no BDNF
Banaschewski et al., 2010 [38]	Review	ADHD genetics populations; enuresis not primary focus	N/A	Frontostriatal and frontocerebellar circuits implicated in ADHD; molecular genetics overview identifies brain regions and pathways where BDNF acts	Review format; genetic focus; no enuresis or direct BDNF measurement
Cho et al., 2010 [39]	Genetic association study; ADHD children vs. controls	Children with ADHD; enuresis not studied	BDNF gene analysis (no serum/plasma)	Sex-specific modulation of *BDNF* gene in ADHD; estrogen modulates cortical BDNF protein functionality	Genetic association only; replication needed; no enuresis arm; no peripheral BDNF measurement
Tsai, 2007 [6] †	Hypothesis/commentary	ADHD populations (theoretical); no direct enuresis data	N/A (theoretical)	Proposes decreased central BDNF activity as primary or secondary feature of ADHD; supports role of dopamine system; mechanistic framework for BDNF–ADHD link	Hypothesis paper; no original data; speculative; borderline exclusion criterion
**Section B—ADHD and BDNF (n = 6)**					
El-Saied et al., 2024 [36]	Cross-sectional clinical study; moderate sample	Children with ADHD vs. healthy controls; enuresis mentioned contextually	Serum BDNF, proBDNF, proBDNF/BDNF ratio	proBDNF/BDNF ratio associated with EEG abnormalities and cognitive severity in ADHD; isoform distinction clinically relevant	Cross-sectional; moderate sample; enuresis not a primary outcome; potential citation misapplication for IQ/cognitive claims
Traver et al., 2006 [9] †	In vitro/animal experimental study	Locus coeruleus noradrenergic neurons (rat); not pediatric clinical	N/A (cellular/molecular)	BDNF mediates phenotypic differentiation of LC noradrenergic neurons; CRF enhances this via cAMP pathway; directly links BDNF to LC—key structure in both ADHD and enuresis	Animal/in vitro study; borderline exclusion criterion; retained for mechanistic centrality to LC–BDNF–noradrenaline pathway
Shim et al., 2008 [5]	Cross-sectional clinical study; small N	Children with ADHD vs. healthy controls; enuresis not studied	Plasma BDNF	Increased plasma BDNF in ADHD children vs. controls; compensatory mechanism via dopaminergic/serotonergic dysregulation hypothesized; positive correlation with inattention severity	Small sample; cross-sectional; no enuresis arm; plasma compartment only
El Ghamry et al., 2021 [40]	Cross-sectional clinical study; Egyptian children with ADHD	Children with ADHD vs. controls; enuresis not studied	Plasma BDNF	Elevated plasma BDNF in ADHD; levels correlate with symptom severity including inattention dimension	Single population (Egyptian); cross-sectional; no enuresis; possible ethnic specificity limits generalizability
Scassellati et al., 2014 [41]	Cross-sectional clinical study; N = drug-naïve children with ADHD vs. controls	Drug-naïve children with ADHD vs. healthy controls; enuresis not studied	Serum BDNF	No significant difference in serum BDNF in drug-naïve children with ADHD; heterogeneity in ADHD pathophysiology and sampling methods discussed	Small sample; cross-sectional; drug-naïve only; no enuresis
Cubero-Millán et al., 2017 [42]	Longitudinal clinical study; N = children with ADHD vs. controls	Children with ADHD on methylphenidate vs. controls; enuresis not studied	Serum BDNF	Daily BDNF fluctuations differ in ADHD; methylphenidate response varies; no relationship with depressive symptomatology	Small sample despite longitudinal design; medicated patients included; no enuresis arm
**Section C—BDNF and Enuresis: Direct Studies and Foundational BDNF Biology (n = 15)**					
Lommatzsch et al., 1999 [11]	Original research (adult tissue); N = adult visceral tissue samples	Adult visceral epithelia including bladder; not pediatric enuresis	Tissue BDNF	BDNF produced abundantly by visceral epithelia; bladder BDNF levels 15× higher than brain parenchyma; local paracrine functions proposed	Adult tissue; not pediatric; no enuresis diagnosis; foundational evidence only
Ochodnicky et al., 2012 [12]	Review	Bladder dysfunction populations (mixed); enuresis not primary condition	Urinary and tissue BDNF (review)	Elevated neurotrophins in bladder dysfunction (spinal cord injury, inflammation, detrusor overactivity) reflect neuroplastic changes in sensory afferents	Review format; mixed populations; no pediatric enuresis-specific data; mechanisms proposed not directly tested
Wang et al., 2014 [28]	Clinical study; N = adult females with overactive bladder	Adult females with overactive bladder; not pediatric enuresis	Urinary BDNF	Urinary BDNF elevated in females with overactive bladder; proposed as objective diagnostic biomarker; more sensitive than urinary NGF	Adult female with overactive bladder; not pediatric; no ADHD; limited direct applicability to enuresis triad
Ece et al., 2019 [7]	Genetic and clinical study; N = children with PMNE vs. controls (small sample)	Children with primary MNE; MNE subtype	Urinary BDNF and NGF; *BDNF*/*NGF* gene polymorphisms	Higher urine BDNF and NGF in PMNE vs. controls; *BDNF*/*NGF* gene polymorphisms examined; elevated neurotrophins may reflect delayed neuromaturation or increased sensory nerve excitability	Small sample; cross-sectional; no ADHD arm; urinary BDNF reflects local bladder production, not central BDNF activity
Morizawa et al., 2019 [29]	Clinical study; N = children with MNE vs. controls (small sample)	Children with MNE; MNE subtype	Urinary NGF/Cr and BDNF/Cr ratios	Urinary NGF/Cr and BDNF/Cr ratios significantly higher in MNE vs. healthy controls; urinary NGF/Cr may predict poor treatment response to standard therapy	Small sample; no ADHD arm; predictive value requires validation in larger cohorts; urinary measure reflects local bladder activity
Barde et al., 1982 [30]	Original research (animal/tissue); seminal paper	Pig brain tissue; not clinical pediatric	Tissue BDNF (purification)	First purification and characterization of BDNF from mammalian brain; established BDNF as distinct from NGF	Animal/tissue study; seminal foundational reference; no clinical pediatric data
Foltran & Diaz, 2016 [26]	Review	Neurogenesis/serotonin model populations; not clinical pediatric	N/A (mechanistic review)	proBDNF and mBDNF isoform biology reviewed; role in neurogenesis, serotonin, and learning and memory pathways	Review format; no original data; no pediatric or enuresis/ADHD data
Colucci-D’Amato et al., 2020 [3]	Review	Depression, neurodegeneration, brain cancer models; not pediatric enuresis/ADHD	N/A (mechanistic review)	Comprehensive BDNF physiology review; TrkB and p75NTR signaling; role in hippocampal neurogenesis and synaptic plasticity	Review format; no original pediatric data; background reference only
Pezawas et al., 2004 [31]	Neuroimaging genetic study; N = adults	Adult humans with BDNF Val66Met polymorphism	N/A (neuroimaging)	BDNF Val66Met polymorphism associated with variation in prefrontal cortical volume in humans; gene on chromosome 11p13-14	Adult study; neuroimaging methodology; no pediatric or enuresis/ADHD data
Pan et al., 1998 [32]	Animal/pharmacology study	Rat blood-brain barrier model	N/A (BBB transport study)	BDNF crosses the blood-brain barrier bidirectionally; efflux rate simulates CSF reabsorption; brain and serum fluctuations generally congruent	Animal model; not human pediatric; foundational pharmacokinetic reference
Radka et al., 1996 [33]	Immunoassay development study	Human and rat serum/brain samples; not pediatric clinical	Serum BDNF (immunoassay)	Serum BDNF ~20× more concentrated than plasma; two compartments not correlated; sensitive immunoassay developed for BDNF detection	Methodological paper; dated (1996); not pediatric or clinical; foundational for BDNF measurement methodology
Lommatzsch et al., 2005 [34]	Cross-sectional study; N = adult humans	Adults across age/weight/sex groups; not pediatric	Platelet and plasma BDNF	Plasma BDNF correlates with platelet BDNF levels; age, weight, and sex influence BDNF levels	Adult study; not pediatric; no enuresis or ADHD; methodological reference for BDNF compartments
Karege et al., 2002 [35]	Cross-sectional clinical study; N = adult patients	Adults with major depression vs. controls; not pediatric	Serum BDNF	Decreased serum BDNF in adults with depression; no correlation with depression severity	Adult depression population; not pediatric; cited for context only—findings should not be extrapolated to ADHD or enuresis in children
Borodinova & Salozhin, 2017 [27]	Review	CNS animal and human models; not clinical pediatric	N/A (mechanistic review)	proBDNF promotes apoptosis via p75NTR; mBDNF promotes survival via TrkB; equilibrium between isoforms determines CNS activity; most studies measure total BDNF without distinguishing isoforms	Review format; no original clinical data; isoform distinction critical for interpreting clinical studies
Thoenen, 1995 [4]	Review	Neuroplasticity models; not clinical pediatric	N/A (mechanistic review)	Neurotrophins including BDNF regulate synaptic plasticity, long-term potentiation, and learning; foundational neurotrophin biology	Seminal review; no original clinical data; background foundational reference

Studies examining ADHD–enuresis, ADHD–BDNF, and BDNF–enuresis relationships are presented in separate sections. Foundational BDNF biology references are included in Section C to contextualize findings on BDNF compartments, isoforms, and measurement methodology. † Borderline inclusion: Koff 1996 [8] (pre-2000 commentary, exclusion criterion) and Tsai 2007 [6] (hypothesis paper, exclusion criterion) were retained for their mechanistic centrality to the review argument. Traver et al. 2006 [9] (animal/in vitro, exclusion criterion) was retained as it directly links BDNF to the locus coeruleus, a structure central to both ADHD and enuresis pathophysiology. N/A = not applicable. BDNF compartments: serum, plasma, urinary, tissue, or N/A. Enuresis subtypes: MNE = monosymptomatic nocturnal enuresis; DUI = daytime urinary incontinence.
